# Biopsychosocial Functions of Human Walking and Adherence to Behaviourally Demanding Belief Systems: A Narrative Review

**DOI:** 10.3389/fpsyg.2021.654122

**Published:** 2021-08-04

**Authors:** Shane O’Mara

**Affiliations:** School of Psychology and Institute of Neuroscience, Trinity College Dublin, University of Dublin, Dublin, Ireland

**Keywords:** walking, imagination, evolution, idealized, pilgrims and pilgrimage

## Abstract

Human walking is a socially embedded and shaped biological adaptation: it frees our hands, makes our minds mobile, and is deeply health promoting. Yet, today, physical inactivity is an unsolved, major public health problem. However, globally, tens of millions of people annually undertake ancient, significant and enduring traditions of physiologically and psychologically arduous walks (pilgrimages) of days-to-weeks extent. Pilgrim walking is a significant human activity requiring weighty commitments of time, action and belief, as well as community support. Paradoxically, human walking is most studied on treadmills, not ‘*in the wild’*, while mechanistically vital, treadmill studies of walking cannot, in principle, address why humans walk extraordinary distances together to demonstrate their adherence to a behaviourally demanding belief system.

Pilgrim walkers provide a rich ‘living laboratory’ bridging humanistic inquiries, to progressive theoretical and empirical investigations of human walking arising from a behaviourally demanding belief system. Pilgrims vary demographically and undertake arduous journeys on precisely mapped routes of tracked, titrated doses and durations on terrain of varying difficulty, allowing investigations from molecular to cultural levels of analysis. Using the reciprocal perspectives of ‘*inside→out*’ (where processes within brain and body initiate, support and entrain movement) and ‘*outside→in*’ (where processes in the world beyond brain and body drive activity *within* brain and body), we examine how pilgrim walking might shape personal, social and transcendental processes, revealing potential mechanisms supporting the body and brain in motion, to how pilgrim walking might offer policy solutions for physical inactivity.

## Introduction

Human walking is a socially embedded and socially moderated biological adaptation, conferring on us a singular upright posture, with a mobile head and eyes atop the spinal column (O’Mara, 2019). Bipedality frees our hands for gesture, tool use, food- and child carrying, among many other functions. We humans have a remarkable range on foot; engaging in regular, sustained walking is profoundly health promoting. Human walking is also social, and social walking is demonstrative: our walking with others signals to yet others our participation in shared intentions and collective goals. We humans walk together to demonstrate adherence to behaviourally demanding belief systems; to source food we will share; for social display; to try and change the world; we walk together to find better lives for ourselves and for each other. Across the world, tens of millions of humans undertake walking journeys – pilgrimages – often of substantial lengths, in the service of goals involving substantial commitments of time, action and belief, as well as community support and involvement. Here, we argue that, while relatively neglected theoretically and empirically, pilgrim walkers can comprise a ‘living laboratory’ bridging literary, historical and religious inquiries, to progressive theoretical and empirical investigations of human walking undertaken because of adherence to a behaviourally demanding belief system. Pilgrim walkers offer advantages for causal and mechanistic investigations: pilgrims vary demographically and undertake arduous journeys on precisely mapped routes of tracked, titrated doses and durations on terrain of varying difficulty, in groups of differing sizes with varying social support, differing belief systems and differing levels of motivation. An organising framework, adopting the reciprocal perspectives of ‘*inside→out*’, where processes within brain and body initiate, support and entrain movement, and ‘*outside→in*’, where processes in the world beyond brain and body drive activity *within* brain and body, is used to integrate molecular-physiological to sociocultural levels of analysis. Situating pilgrim walking in a naturalistic, evolutionary and biopsychosocial context makes it a fruitful subject of empirical enquiry and may also offer implications for correcting some of the malign effects of physical inactivity in many societies.

## Human Walking and Human Dispersion

The evolution of human walking has been intensively investigated from a variety of perspectives (e.g. Schmitt, 2003; Addis et al., 2017; Senut et al., 2018), with fossil evidence of hominid-like bipedal walking emerging from 3 to 8 mya (Lovejoy, 2016). Comparative investigations of walking energetics reveal, for example, that humans travel about twice as far on foot, calorie for calorie, as our nearest primate relatives (Sockol et al., 2007; Pontzer et al., 2009). From about 60–180 kya (Bae et al., 2017; Hershkovitz et al., 2018), humans made multiple journeys on foot out of Africa to the Eurasian landmass, eventually dispersing to the Americas and the greater Asia-Pacific region (López et al., 2015), slowly populating the planet. This journey was undertaken, in migratory groups, in multiple waves, over thousands of years, populating the planet by walking together in migratory families, tribes and other groups. Humans achieved global dispersion because of our adaptations as social walkers (O’Mara, 2019): our remarkable geographical distribution makes us unlike any other species, for no other species has spread around the world in the promiscuous fashion we humans have. Selection effects binding individuals to their groups in the service of shared goals must have been paramount during these migratory walks of families, tribes and other groups (Hamilton, 1964; Mullon et al., 2018); trace fossil evidence of human group migration supports this claim (Liutkus-Pierce et al., 2016), as do the forms of alloparenting and reproduction practiced in human groups (Hrdy, 2009, 2017; Kenkel et al., 2017). Shared goals might be day-to-day survival (food sourcing, avoiding predation, finding safe shelter and the like) or longer term (such as migrating to places that humans had not been to before and are imagined to be better than current circumstances).

## Human Walking From an ‘*Inside→Out*’ Perspective: Preliminary Considerations

Studies of human walking are dominated by laboratory treadmill studies (Cronin and Finni, 2013), with experiments mostly focused on acute, rather than endurance, performance (e.g. Contrepois et al., 2020); treadmill studies are logistically easier to conduct, requiring only one or two short (~1–2 h) laboratory visits. Long-term exercise studies are more easily conducted in small animal models (especially the rat: e.g. Oliff et al., 1998; Basso and Suzuki, 2017; Callaghan et al., 2017). *Such studies have revealed the spinal and other* mechanisms supporting locomotion; changes entrained in body and brain by locomotion; and molecular, cellular and systems changes occurred because of locomotion. Walking involves coordinated actions by widespread brain regions supporting decision-making, goal-seeking, memory and planning, and of motor and spinal regions responsible for motor pattern generation and, eventually, of muscle groups (synergies) (Bizzi and Cheung, 2013; Flash and Bizzi, 2016) entraining movement. Movement feeds back on to brain and bodily function: for example, regular, daily bouts of brisk walking (at 70–80% of predicted maximal heart rate) enhances cardiovascular health (Murphy et al., 2002); higher daily step counts are associated with substantially reduced all-cause mortality (Saint-Maurice et al., 2020); aerobic fitness supports neurocognitive function throughout the life course (Ludyga et al., 2020). Finally, there is evidence that walking programmes may assist in vascular and neuro-rehabilitation, where deficits in walking are absent. Supervised walking therapy helps markedly with the painful constriction of blood vessels (‘claudication’) in the legs, arising from circulatory problems (Fakhry et al., 2012). It is a reasonable surmise that supervised walking might also assist rehabilitation with acquired brain injury, depending on the nature, type and extent of injury – perhaps by promoting blood flow, by entraining electrical rhythms in the brain and by engaging cognitive processes through systematic dual tasking (talking and walking; recall of learning information and walking; etc.). This idea has not been tested in any great depth, but evidence (Evans et al., 2009) suggests at least some improvements in cognition arising from having people walk and perform a cognitive task simultaneously (see also Fritz et al., 2015; Lauenroth et al., 2016).

## Malign Consequences of Physical Inactivity

Selection effects have ensured regular, physical activity is profoundly health promoting in humans (Raichlen and Alexander, 2017): the average 80yo Tsimane in the Amazonian jungle, for example, living a non-mechanised ‘ancestral’ lifestyle, walks everywhere and has a cardiac health equivalent of an average Westerner 25 years younger (Kaplan et al., 2017). Humans must balance two opposing metabolic drives: food sourcing (for energy) and energy conservation (Lieberman, 2015). Historically, these drives have rarely been in balance, with food sources rare, and energy conservation at a premium. Nineteenth-century day labourers in London, for example, typically walked ~6–8 miles/day (~9–13 km, to and from work (Hobsbawn, 1964; Clayton and Rowbotham, 2009). In the twenty-first century, cheap calories are plentiful, but we have engineered movement out of our daily working and leisure lives (Biswas et al., 2015), with adults in high-income countries walking typically ≤4–5 k steps/day (3–4 km; Althoff et al., 2017). Walking <5 k/steps a day is defined physiologically as sedentariness, bringing a correlated surge of non-communicable diseases, ranging from type two diabetes to major depressive disorder (Tremblay et al., 2010; Tudor-Locke et al., 2013). The World Health Organisation and other agencies have repeatedly concluded rising physical inactivity levels (Peçanha et al., 2020), diseases of aging, and, of metabolism, are a major problem in many societies, all of which are exacerbated as the world’s population ages (Villareal et al., 2005; Jin et al., 2015). Many diseases of aging are preventable through prophylactic measures (Booth et al., 2011; Reynolds et al., 2019), especially regular exercise: active lifestyles are universally recommended for their health-promoting properties and for preventing non-communicable diseases (even major depressive disorder; Harvey et al., 2018) and diseases of ageing (Goryakin et al., 2019; Savikangas et al., 2020). Physical activity can abrogate pathologies of cognition accompanying ageing (e.g. Livingston et al., 2017, 2020); consequently, the neuroscientific consensus concurs with active lifestyle advice: exercise benefits *all neurocognitive domains* (from memory, executive function and beyond; Ludyga et al., 2020).

## Common Neurocognitive Substrates Support Cognitive Mapping, Memory and Imagination

Experiments confirm recent suggestions (Keinänen, 2016; O’Mara, 2019) that walking may drive creative idea production and facilitate imaginative thinking (Oppezzo and Schwartz, 2014; Keinänen, 2016; Kuo and Yeh, 2016). Oppezzo and Schwartz (2014) show short periods of corridor or treadmill walking approximately doubles creative idea production in a divergent thinking task, compared with a similar seated period; Kuo and Yeh (2016) find a similar effect in aging adults, where a short period of walking approximately doubles their creative idea production, compared to young seated controls. The underlying neurocognitive mechanisms have not been fully delineated, but whole-body (locomotor) movement drives hippocampal formation activity to a remarkable degree (e.g. O’Mara et al., 1994; Czurkó et al., 1999; Aghajan et al., 2017; Hazama and Tamura, 2019), probably *via* vestibular stimulation (Vitte et al., 1996; Suzuki et al., 2001), thereby amplifying functions supported by the hippocampal formation (such as cognitive mapping and memory). Contrariwise, persons with amnesia arising from hippocampal damage have impoverished imaginations, mind wandering and dreaming, while also being impaired in spatial orientation and navigation (Zeidman and Maguire, 2016; McCormick et al., 2018; Spanò et al., 2020). Thus, in a remarkable theoretical and empirical convergence, the neurocognitive systems active during memory and imagination are also largely the same systems active during whole-body movement: cognitive mapping, memory, mental time travel and imagination, all sharing common neural substrates in the extended hippocampal formation (Hassabis and Maguire, 2007; Buckner, 2010; Irish et al., 2011, 2013; O’Mara and Aggleton, 2019).

A provocative hypothesis is that walking may affect autobiographical memory construction and recall. The ‘*default-mode*’ of brain activity (Raichle, 2015) occupies c. 40% of our walking hours and involves mental time travel – coursing backwards and forwards through both the ‘big picture’ *and* the details of our lives, allowing us to construct personal autobiographical narratives. ‘Constructing narratives’ about one’s self and one’s social world is a key function of default-mode processing (Buckner, 2010; McAdams, 2013; McAdams and Guo, 2015). Through narrative construction, we can integrate our experiential and remembering selves, forming a coherent, agentic and adaptive identity. O’Mara (2019) suggests regular and sustained walking facilitates the mental time travel central to creating personal narratives – constructing our autobiographical stories and the meaning of the wider (social) world within which we live. Thus, walking, on this view, facilitates activity in the brain networks processing memory and meaning, in part, because whole-body locomotor movement activates the hippocampal formation and related structures and, in part, because information sharing during social walking readily enters into our individual memories (Adolph et al., 2008; Webb et al., 2017; O’Mara, 2019). Gibson and Nicholas (2018), for example, find social walking assists in autobiographical narrative construction of shared experiences and the construction of tactile autobiographical memories in persons with dual sensory loss (congenital deaf-blindness).

## Human Walking From an ‘*Outside→in*’ Perspective: Preliminary Considerations

Hearing others walking activates brain networks supporting social cognition (Saarela and Hari, 2008; Arioli and Canessa, 2019), emphasising how the sound of others’ footfalls enters social cognition. Walking with others makes demands on many brain and behavioural systems supporting the directed, coherent movement of the group towards some goal and of group survival (Raafat et al., 2009; O’Mara, 2019; Shamay-Tsoory et al., 2019). Thus, we have brain systems and subsystems tuned to interpersonal synchronisation and pacing of walking movements (Noy et al., 2017; Soczawa-Stronczyk et al., 2019); wayfinding and cognitive mapping (O’keefe and Nadel, 1978; O’Mara and Aggleton, 2019); environmental vigilance and threat detection (Eilam et al., 2011); goal orientation and achievement (Dolan and Dayan, 2013); shared social attention and gesture (Redcay et al., 2016); tolerating uncertainty (Peters et al., 2017); optimism bias and risk-taking (Dricu et al., 2020); memory and motivation (Aggleton et al., 2010; Chiew et al., 2018; O’Reilly, 2020); imagination (Beaty et al., 2016); and more. Walking with others, on this view, amplifies these processes through shared storytelling and interpersonal synchronisation during the movement of the group towards a common, and perhaps imagined, destination. This view suggests, therefore, intrapersonal processes support interpersonal transactions with the environment (Baumeister, 2016).

## Human Pilgrim Walking as Transient Mass Migration

Annual pilgrimages are possibly the largest form of transient, human mass migrations (Wilder-Smith, 2006): worldwide, Arcworld (2015) estimates 155 M people annually engage in major pilgrimages (Griffin and Raj, 2017), with an estimated 30 M pilgrims attending the *Ayyappan Saranam* festival in India (Hindu), and 20 M attending the *Our Lady of Guadalupe* pilgrimage (Christian) in Mexico (PILGRIMAGE STATISTICS-ANNUAL FIGURES, n.d.). About 2.5 M attended the *Hajj* in Mecca in 2019 (Hajj statistics, 2019). These pilgrimages involve combinations of substantial walking and mass transport, because individual vehicular travel is often impossible, given the density of people and lack of road space. Many hundreds of thousands annually walk the pilgrim walking trails in Europe (which run to a cumulative many thousands of kilometres in length). The most walked pilgrimage in Europe is the *Camino de Santiago* in Galicia (Lois-González et al., 2018); the most popular route is the *Camino Francés* (780 km: St Jean-Pied-du-Port, Biarritz to Santiago); the longest starts at Seville and is 1,200 km. Some 327,378 pilgrim walkers received the Pilgrim’s Certificate (*Compostela*) from the Pilgrim’s Office in 2019, an underestimate of the numbers walking these routes, as many do not claim the certificate, or walk for non-religious, secular purposes (Oviedo et al., 2014; Amaro et al., 2018). These ancient traditions of physically and psychologically arduous pilgrimage walks are of days-to-weeks duration and are undertaken for secular and/or religious reasons. Pilgrimages are a notable example of social walking, even when performed by a solitary pilgrim. Pilgrimages are performed in solidarity with a greater purpose – a community, a cause and a faith, demonstrating the power of a behaviourally demanding belief system to animate a walking tribute to that belief system. Even the solitary pilgrim is walking for, and with, an imagined community of the mind. (*Note:* ‘pilgrim walking’ here embraces religious and secular walkers walking these ancient routes.)

## Pilgrim Walking as a Literary, Social and Contemporary Phenomenon

The earliest-recorded walking pilgrimages date to >5–10 kya (Gitlitz et al., 2002; Murphy, 2012). Pilgrimages are celebrated, discussed and analysed in numerous important literary, historical and religious works. Geoffrey Chaucer’s fourteenth-century work, ‘*The Canterbury Tales*’, for example, concerns a pilgrimage from London to Canterbury; one character, the ‘Wife of Bath’, is described as ‘wandr’ring by the Way’, having undertaken what would have been very arduous pilgrimages to Jerusalem, Rome and ‘Galice’ (Galicia: Santiago di Compostela), Bologna and Cologne. Pilgrim walking is a metaphoric device in other works: in the ‘*Inferno*’, Dante the poet is also Dante the pilgrim, journeying the Circles of Hell; Bunyan’s ‘*Pilgrim’s Progress’* is written as a dream sequence, where the pilgrim walks from the ‘slough of despond’ to the ‘celestial city’. More recently, Cormac McCarthy’s ash- and death-strewn novel, ‘*The Road*’, is a pilgrimage of necessity by the unnamed father and son, while they are ‘carrying the fire’, walking the road to, perhaps, nowhere.

## Pilgrim Walking as a ‘Living Laboratory’ – From ‘Inside→Out’ to ‘Outside→in’

Pilgrimage walking is empirically underexplored; interest most often focuses on anthropological, tourist economics, or crowd and leisure management aspects of pilgrimage (Courtney, 2015). In principle, pilgrim walkers (PWs) lend themselves to controlled investigations: consider the possible effects of a 780 km walk undertaken over 42 days (~18.5 km/day). The physiometabolic effects of a such a walk can be investigated through testing factors in blood, measures of cardiac and aerobic fitness, and changes in visceral fat deposition. Similarly, changes in social interactions or social affiliation can, in principle, be tested by measuring entrainment of interpersonal interactions with other members of the walking group (perhaps *via* experience sampling methodologies). Thus, pilgrim walkers can provide a fertile ‘*living laboratory*’, bridging literary, historical and religious inquiries, to progressive theoretical and empirical investigations in a controlled ‘model system’, combining ease of investigation with generalisability. Pilgrim walkers (PWs) are homogenous with respect to intervention and experimental control: PWs undertake walks of titrated doses and durations (Camino walkers – up to 1,200 km/10 weeks), with metabolic demands requiring an approximate doubling of daily caloric intake (Harris, 2019). Moreover, PWs quickly adapt to these extended walks, whereas runners may take many months training for single-occasion endurance events (such as marathons of 42.2 km/4–5 h duration). PWs are a large and demographically variable population running from about 5 ya to the late 1980s, of various aerobic statuses and health conditions, with a marked representation of those in middle age and above. Further, some PWs are persons with neurological conditions (e.g. stroke), other underlying health conditions (e.g. cardiovascular disease; diabetes; and frailty), sensory or motor impairments (visual, auditory, etc.), neuropsychiatric conditions and mobility impairments requiring movement aids (walking sticks or crutches, prosthetic limbs or all-terrain wheelchairs). PWs undertake walks for secular (e.g. health; challenge; and bucket list) and/or religious (e.g. penitential and obligatory) purposes. Some walk for ‘self-healing’ or ‘self-therapy’ (Egan, 2010; Jørgensen et al., 2020), a notable feature of walking groups practicing ‘shared or communal therapeutic mobility’ (Pollard et al., 2020). Pilgrim routes are geospatially mapped (and, *via* smartphone, digitally tracked), of varying terrain and difficulty, testing individual differences in engagement, motivation and strain. Finally, while walking can be central to our social lives, correlation and causation cannot be untangled easily: for example, a study of the elderly concluded those walking about 150 min/week are more socially active with greater overall wellbeing than those walking less (Donoghue et al., 2016). Which direction does causality run? Do you walk more because you are socially active, or are you socially active because you walk more? PWs offer a way to untangle correlation and causation, with implications for a key problem for behavioural change, viz., countering the general reduction in physical activity apparent across the world.

## A Biopsychosocial Framework for Investigating Pilgrim Walking

Laboratory, small-animal and human treadmill-based studies have been and will continue to be, mechanistically vital, but cannot, in principle, address why humans walk substantial distances together for behaviourally demanding belief systems. Here, we offer a naturalistic framework for investigating pilgrim walking and, in turn, attempt to derive some policy prescriptions from this population to engineer physical activity into our lives. The overall framework aims to situate pilgrim brain and walking body in a biopsychosocial context, but moving away from the usual ‘top-down’ and ‘bottom-up’ cognitive processing dichotomy (Awh et al., 2012; Ramsey and Ward, 2020) in favour of the reciprocal perspectives of ‘*inside→out*’ (the ‘world’ within the walker), where processes within brain and body initiate, support and entrain movement, and those from ‘*outside→in*’ (the walker within the world), where processes in the world beyond brain and body drive activity *within* brain and body. Note, the *inside→out*’ case, predicts, for example, there should be widespread changes evident in brain and end-organ systems arising from physical activity (for example, increases in circulating factors, such as neurotrophins, supporting structural change in the brain in those participating in a physical activity programme and also enhanced performance in hippocampal-dependent memory function (as is the case; Griffin et al., 2011). However, the latter, ‘*outside→in*’, case suggests body and brain are embedded in a physical and sociocultural context, where sensory transduction occurs through well-delineated sensory channels, but the information processed is various, embracing cognitively abstract entities (such as social structures), to more immediate sensory processes (such as the texture and firmness of the ground beneath the feet). These processes require intrinsic (i.e., brain region→brain region) activity entrained by multiple brain networks supporting action-oriented processes, co-opted both to spatio-physical demands and to cognitively abstract ends – for example, goals as particular physical places and goals as socially determined achievements (or both).

**Figure 1:**
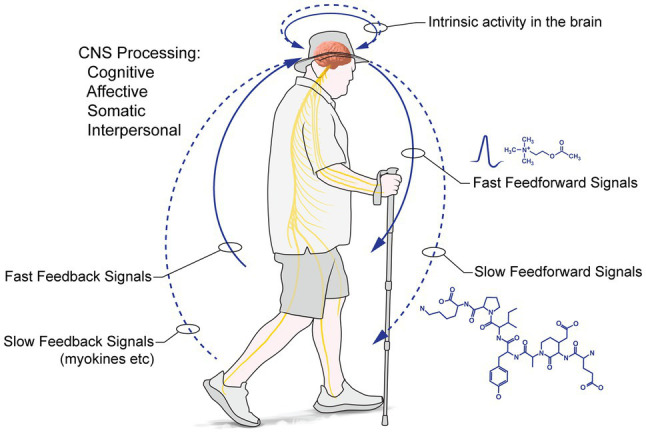
Personal: PW entrains reciprocal, feedforward (brain→body), feedback (body→brain) and intrinsic (brain region→brain region) intrapersonal processes supporting and consolidating changes in brain and body.

*From inside→out: Intrapersonal processes support interpersonal transactions with the environment:* PW entrains intrapersonal processing (Figure 1): these intrapersonal processes are reciprocal, feedforward (*brain→body*), feedback (*body→brain*) and intrinsic (*brain region→brain region*), supporting and consolidating changes in brain and body. We can also distinguish fast and slow actions (mediated *via* neurotransmission and neurohumorally, respectively). The effects of walking are dose- and context-dependent, with greater walking doses of greater effect, depending on the organ system and function (changes in cardiovascular factors or functional connectivity of the hippocampal formation, for example, will show greater effects for longer distances, eventually asymptoting; moment-to-moment mood may reflect transient variables, but underlying affect should show a dose-dependent, positive augmentation). These intrapersonal processes are further moderated by social and transcendent processes (discussed below).

*A specific example:* cognitive mapping and wayfinding, supported by hippocampal formation activity, require sensory input, motor output and intrinsic activity within, and between, differing brain regions. Feedforward processes entraining brain regions initiate and control destination-directed walking through the environment. Fast feedback loops arising from the vestibular system stabilise movement; cognitive processing of sensory inputs arising from the movement of other walkers allows the prediction of the trajectory of other walkers, enabling collision avoidance, or following, as appropriate (Sweeny et al., 2013). Transportable factors result from activity: for example, sustained, contractile muscle activity produces myokines (muscle-secreted molecules; Pedersen, 2019), such as skeletal myofiber vascular endothelial growth factor – smVEGF (Rich et al., 2017), which diffuse through the vasculature, changing and enhancing the fabric and functions of myriad brain and body regions. We predict positive changes in pro- and anti-inflammatory factors in blood, as well as sustained expression of certain myokines/factors secreted in blood. Several studies support these predictions. In an endurance-walking case study, Ardigo et al. (2011) investigated a single 62-year-old male walking 1,300 km/68 days over the *via Alpina*. He adapted quickly and easily to breathing at high altitudes; his body mass index declined by c. 10%; measured body fat fell c. 25%; and there was a c. 75% decrease in the triglycerides thought to underlie some forms of cardiovascular disease and a converse increase in cardioprotective high-density lipoproteins (see also Pedrinolla et al., 2018), underscoring our ready adaptation to endurance walking. Thus, prolonged hiking in nature is something humans adapt readily to, and the physiological changes arising likely share great commonalities with those found in PWs. In this regard, Bemelmans et al. (2010, 2012) investigated 29 healthy males and females (40–70 ya) undertaking at least 280 km of the Santiago di Compostela, concluding there are immediate and positive impacts on the main cardiovascular risk factors because of the strenuous nature of PW (and also perhaps because of some dietary changes and caloric burn); furthermore, higher walking speeds have a more positive impact on cardiovascular health (Harris and Wolf, 2013). Finally, those living ‘ancestral’ hunter-gatherer lives today, regularly, walk ~10–15 km/day and have remarkable cardiac health (Pontzer et al., 2012; Kaplan et al., 2017), which disimproves when they adopt a sedentary lifestyle (Dounias and Froment, 2006; Dounias et al., 2007).

**Figure 2:**
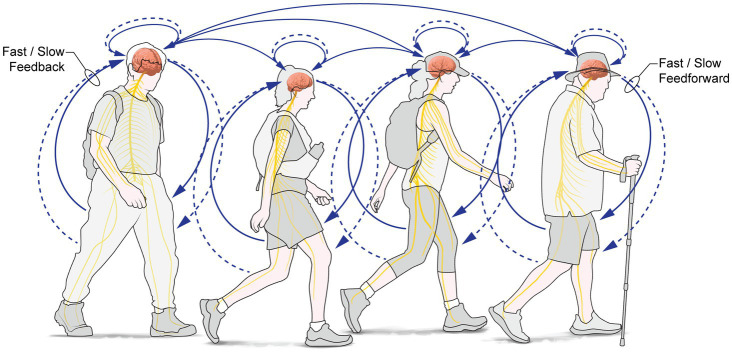
Social: During PW, intrapersonal processes reciprocally support interpersonal social connectedness, social relationships and social synchronisation (with those physically or psychologically or both, present); these are built on underpinning, consolidating and supporting changes in brain and body.

*From outside→in: Intrapersonal processes reciprocally support and entrain interpersonal and social interactions:* During PW, intrapersonal processes reciprocally support interpersonal social connectedness, relationships and synchronisation (with those physically, or psychologically, present or both); these are built on underpinning, consolidating and supporting changes in brain and body (Figure 2), because most ‘inner processes serve interpersonal functions’ (Tice and Baumeister, 2001), such as inferring mental states, intentions and agency of others. Affiliation to a group requires minimal experience with that group (Baumeister and Leary, 1995): affiliation happens quickly, and subsequent experience supports the continued expression of group affiliation, even to an arbitrarily assigned group (Dunham, 2018), such as a tour group, for example.

*A specific example:* Cognitive mapping and wayfinding are co-opted to serve interpersonal functions: walking groups must find their way to a goal and coordinate individual decision-making processes of group members to do so. Goal-finding by a group requires the group leverages individual processes to select a/the goal (the eventual destination) of the group. Joint information sharing, joint decision-making, and consensus-finding entrains inner individual processes as the group decides on the destination, and the subgoals to be achieved during wayfinding to the destination. Ensuring the coherent and directed movement of the group requires individuals attend to each other’s movements, synchronising their walking so it is directionally and goal oriented. As the group grows larger, coordination is more easily achieved by singing, chanting, recitations or music-making, among other means.

*From outside→in and inside→out: Transcendent processes are iterative – driven in part by pre-existing personal traits, but subject to negative and positive feedback from the journey undertaken:* PW is undertaken for secular and/or religious reasons, particularly of religious feeling, and meaning-making. Underlying self-reported motivations are psychosocial processes, such as ‘meaning-making’ – activities focusing on larger themes in a person’s life. Humans have always sought ‘transcendent’ feelings – for example, by participating in initiation rites or the consumption of psychedelic substances. Secular PW is compatible with transcendent feelings: surveys of PWs (Camino walkers: Oviedo et al., 2014; Amaro et al., 2018) disclose multiple reasons for undertaking the walks, and formal religious purposes are a minority (e.g. a penitential pilgrimage). Pilgrim walking can thus be conceived as a form of ‘*experiential consumption good*’, where the goal is ‘transcendence’, attained by effortfully participating in something from which meaning can be extracted, and where feelings of self and other (people or nature or the universe) are blurred. It is plausible that combining some degree of ‘in-walk’ caloric insufficiency, hypohydration, thermal strain, repetitive, extended, rhythmic, outdoor locomotor activity, prolonged in-group social contact and the psychological tunnelling arising from the extended walk, in combination, gives rise to an occasional sense of psychological dissociation, boundaries of self and other dissolving or even a psychological ‘high’. Finally, activation of endogenous opioid receptor systems arising from physical activity (e.g. Callaghan et al., 2018, 2019) might also contribute in diffuse, analgesic ways. These combined effects might, of course, be misattributed to ‘supernatural’ sources, despite their biopsychosocial origins. This definition of transcendence is empirically testable, in contrast to less-empirically tractable definitions, especially Maslow’s definition (Maslow, 1971), where transcendence is simply classified as ‘…the very highest and most inclusive or holistic levels of human consciousness, behaving and relating’ (p. 269), without specified empirical outcomes.

*A specific example:* Goal-directed behaviour needs not be just to a physical destination: it can be an imagined destination (the imagining of which depends on the activity of the hippocampal formation), or even a sought-after psychological state (such as a perception of ‘one-ness’ with the universe). Religious and secular walkers may find meaning and achievement in finishing the PW, but might attribute the origin of such meaning and achievement to differing sources. MacFarlane (2012; p. 236), for example, surmises individual meaning-making is an important factor in PW, not religious motivations. By contrast, some scholars of religion (MacGregor, 2018) suggest a feeling of religious ecstasy arises on completing the PW for the religious – a point of ‘euphoric climax’ (p. 207). Participatory immanence is the name we give here to the feelings experienced of a ‘supernatural’ manifestation arising from the PW; we extend the concept to the majority non-religious walkers, because feelings of existential communion and universal connectedness may arise in the non-religious also. Certainly, feelings of ‘effervescence’ often arise when an individual is a part of larger group serving a collective purpose (Maheshwari and Singh, 2009; Tewari et al., 2012; Gabriel et al., 2017); effervescent experience includes increases in feelings of wellbeing, lower levels of self-reported loneliness and higher levels of positive feeling and meaning. A ‘euphoric climax’ is certainly possible, but it may be complicated by feelings of relief, or perhaps feelings of loss of immediate purpose, because the journey is now at an end. Degree of self-reported religiosity may, paradoxically, be irrelevant: in a study of atheists walking the Camino, Farias et al. (2019) found religiously committed and atheist pilgrim walker alike were equally committed to exploring transcendent feelings and connectedness to nature, suggesting experience, meaning-making and self-transcendence arise naturally from such extended and arduous walks.

‘*Flow*’ is the subjective experience of concentration and deep enjoyment accompanying or arising from skilled performance (Csikszentmihalyi, 2014), as might arise in extended distance walking (O’Mara, 2019); awe is conceived of as perceiving vastness and attempting to process the experience of the diminution of the significance of the self (Keltner and Haidt, 2003). Such feelings may give rise to the affective changes arising during extended walking. Sturm et al. (2020) find an intervention promoting awe during solo walking of 15 min outdoor walks (1 weekly for 8 weeks), compared to control solo walking, and enhance reported feelings of joy and of prosocial positive emotions. Spillover effects on positive prosocial emotions and decreases in daily distress over time were also apparent. The temporal duration of these effects is uncertain, but regular ‘awe walking’ might prove enduringly beneficial; however, Sturm et al. did not conduct a formal awe walking, dose–response, study to determine the depth and duration of any such putative effects.

## Integration of Personal, Social and Transcendental Processes Into Human Functioning

**Figure 3:**
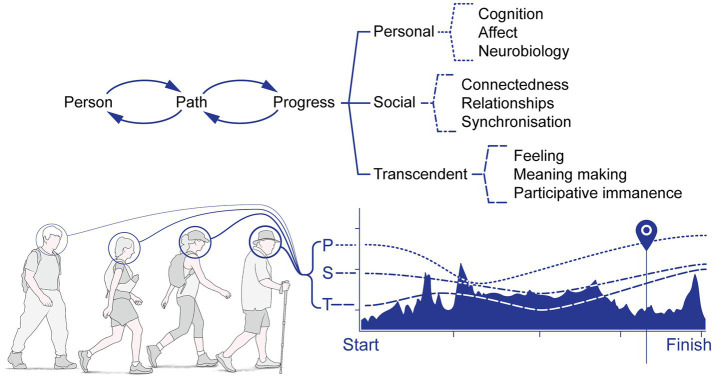
Transcendence: PW is undertaken for secular and/or religious reasons, particularly of feeling, meaning-making and participative immanence. Personal, social and transcendent states may vary across the walking pilgrimage, depending on a variety of factors (such as terrain strain, motivation and feelings towards the group, for example).

We posit the integration of PST processes happens within a reciprocal context: from the ‘*inside→out*’, where the walker avails of the extended walk to create new self-narratives and schemas, supported and consolidated by activity-induced neurobiological modulation of various molecular and organ systems, stabilising and consolidating changes in structure and function of brain, body and beyond, and from the ‘*outside→in*’, because human walking is a socially embedded and socially moderated biological adaptation, entraining and supporting extended movement in particular geographical locales and social contexts (Figure 3).

## Can Lessons From Pilgrim Walking Shape Active Living Throughout the Life Course?

Here, we draw some tentative, and perhaps policy-relevant, lessons from pilgrim walking to foster increases in physical activity, using the ‘*outside→in’* and ‘*inside→out’* perspectives. We know relatively little about how psychosocial mechanisms can facilitate increased physical activity (Stillman et al., 2020); the task is identifying and entraining causal feedback and feedforward psychosocial processes, and employing them to increase activity levels. ‘Social-prescribing’, for example, has been attempted to increase levels of physical activity, but has not yielded large-scale activity increases (Bickerdike et al., 2017; Husk et al., 2020), possibly because social-prescribing efforts are often not theoretically or empirically grounded, or sufficiently embedded in other cultural or policy changes to increase physical activity in society generally.

*From ‘outside→in’:* Public health policy has attempted to increase physical activity levels through advertising campaigns. Such campaigns might consider focusing on the walker’s social environment to provide the additional necessary social motivation, challenge and support, and boost this further by group membership and affiliation. While individual motivation is important, the analysis here suggests membership of extended, social motivational structures supporting regular bouts of individual locomotion is especially important. Increasing levels of walking generally might use formal and informal social walking groups more effectively, perhaps organised through social media, rather than traditional, advertising-based, admonitory, campaigns focused on individual motivation and behavioural change. Social prescribing to increase physical activity might require active group leadership, enrolment, involvement and engagement through social media to be successful. Creating local walking groups with smartphone app route mapping and pedometry capture (British Heart Foundation, n.d.) offers one low-cost method to encourage walking; creating league tables harnessing gentle inter-group competition focused on step counts by teams, harvested by such an app, might be another. Supporting physical activity programmes requires changes in the built environment explicitly supporting socially embedded walking programmes, through safe and extensive pedestrian infrastructure (with adaptations for persons with mobility impairments or other disabilities), and ample urban green space provision; otherwise, such campaigns will not raise physical activity levels, despite public health goals.

Adventitious walking arising as a design by-product of the built environment will assist in increasing physical activity levels. ‘Hard’ and ‘soft’ design components of the built environment are of particular interest. ‘Hard’ changes in the built environment design ensure people engage in active movement as a default of building design (stairs, corridors, walkways and the like), ensuring walking as a default, by de-emphasising passive mobility options (escalators, elevators and the like). In turn, ‘within-building’ walking arises because of environmental design and demands. ‘Green’ designs incorporating aspects of nature also make the built environment more attractive and facilitate walking. ‘Soft’ changes include facilitating active workspaces (which can reduce territoriality and increase creative idea production; Knight and Baer, 2014). Walking desks, as a component of an active workspace, can encourage low-level, consistent walking throughout the day (Schuna et al., 2014), and mimic, in part, the constant demands of pilgrim walking, offering physiometabolic benefits (including weight loss; Levine and Miller, 2007), with benign-to-positive effects on cognitive function (Labonté-LeMoyne et al., 2015; Larson et al., 2015a,b).

*From ‘inside→out’:* Entraining internal processes to increase physical activity levels in parallel with social and environmental supports will augment physical activity programmes. Extending one’s own ‘narrative self’ to include the identity of being a walker will support such programmes (this is a possibility, given the centrality of narrative construction to our overall life course; McAdams, 2019); this is probably best achieved in during social walking, while engaging in discursive exchange with others.

The physical activity involved in walking provides underestimated boosts to affect, even in those who ‘dread’ or dislike walking (Miller and Krizan, 2016), thereby contributing to psychological wellbeing (perhaps in an enduring, dose-dependent way). Pilgrim walking routes often employ rural trails, avoiding the built environment; interestingly, study participants typically markedly underestimate how much walking in nature will boost their experience of both in-walk and post-walk positive affect (Nisbet and Zelenski, 2011). A growing body of evidence points to the importance of nature exposure for mental health (Bratman et al., 2019); although we spend the vast majority of our time indoors (≥85%; Klepeis et al., 2001), we evolved in an outdoor environment, spending most of our evolutionary history in a non-urban setting, where selection effects likely rewarded perception of important environmental signals, such as sources of food, shelter and refuge (Kaplan, 1987; Van der Wal et al., 2013). Positive effects of hiking in nature may arise in part from simple, regular exposure to nature. Moreover, green space exposure through active usage of urban parks can have a marked and positive effect on the diurnal variation in the secretion of the stress hormone, cortisol (Thompson et al., 2012), further supporting positive feedback loops between walking, nature exposure and mental health. A risk factor for major depressive disorder is maladaptive patterns of self-referential thoughts (rumination); short periods (90 min) of walking in nature can substantially reduce rumination, compared to urban-only walks (Bratman et al., 2015). Combining nature/green exposure and experiences using apps with crowd-sourced feedback might therefore be of particular use to boost physical activity.

Pilgrim walking requires regular, structured and diaried performance to successfully complete a lengthy walking route; integrating social media apps with a regular, diaried walking group appointment are now trivial, but may provide a simple, additional mnemonic nudge to increase levels of walking. Walking apps and podcasts could also incorporate prompts for ‘awe walking’; the sustained and regular practice of awe walking might provide necessary support for the ‘psychological immune system’ (Gilbert et al., 1998), again providing an important boost to general mental and physical health.

## Conclusion

Pilgrim walking is an underexplored human behaviour, found in many cultures across the world, with roots deep in our evolutionary past, likely arising from selection effects on both our physiometabolic health and on numerous neurocognitive, social and affective processes, collaterally binding us to our groups and their goals. The reciprocal perspectives of ‘*inside→out*’ (where processes within brain and body initiate, support and entrain movement) and ‘*outside→in*’ (where processes in the world beyond brain and body drive activity *within* brain and body) offer a potentially generalisable biopsychosocial framework to examine how pilgrim walking might shape personal, social and transcendental processes, to how lessons from pilgrim walking might offer solutions for physical inactivity in society. The human urge to engage in, and profit from, extended bouts of walking is best understood in a social context, and as a social behaviour bringing collateral, generally positive, personal changes, which occasionally include inducing transcendental states (the latter best understood as outworkings of biopsychosocial processes of body and brain under rhythmic, physiometabolic strain in a specific social context). Pilgrim walking offers some lessons for augmenting physical activity programmes, through entraining social group membership coupled to individual affiliative processes. Public health goals and urban planning must also meaningfully align for such campaigns to be effective: biomedical and social science advice alike must therefore be incorporated into public policy making addressing physical activity goals (O’Mara, 2019).

## Author Contributions

The author confirms being the sole contributor of this work and has approved it for publication.

## Conflict of Interest

The author declares that the research was conducted in the absence of any commercial or financial relationships that could be construed as a potential conflict of interest.

## Publisher’s Note

All claims expressed in this article are solely those of the authors and do not necessarily represent those of their affiliated organizations, or those of the publisher, the editors and the reviewers. Any product that may be evaluated in this article, or claim that may be made by its manufacturer, is not guaranteed or endorsed by the publisher.
